# Comparison of the kinematics and kinetics of shoulder exercises performed with constant and elastic resistance

**DOI:** 10.1186/s13102-018-0111-7

**Published:** 2018-11-28

**Authors:** Ramona Häberle, Florian Schellenberg, Renate List, Michael Plüss, William R. Taylor, Silvio Lorenzetti

**Affiliations:** 1 Institute for Biomechanics, ETH Zürich, Leopold-Ruzicka-Weg 4, 8093 Zürich, Switzerland; 2 Human Performance Lab, Schulthess Clinic, Lengghalde 2, 8008 Zürich, Switzerland; 3 Swiss Federal Institute of Sport Magglingen, SFISM, Alpenstrasse 18, 2532 Magglingen, Switzerland

**Keywords:** Inverse dynamics, Movement analysis, Load condition, Internal/external rotation

## Abstract

**Background:**

Internal and external rotation exercises of the shoulder are frequently performed to avoid injury and pain. Knowledge about the motion and loadings of the upper extremities during these exercises is crucial for the development of optimal training recommendations. However, a comparison of the angles and corresponding moments in the upper extremities that are achieved during internal and external rotation exercises for the shoulder by using different resistance types has not yet been performed. Therefore, the aim of the study was to examine upper extremity kinetics and kinematics in 3D of the internal and external rotation exercises.

**Methods:**

The kinematics and kinetics of 12 participants while they performed 10 different exercises with a constant and with an elastic external load corresponding to 2% body mass was assessed. The motion of the upper extremities was recorded three-dimensionally with a motion capture system, using a newly developed marker set and joint coordinate systems with 28 markers. The applied external load was measured with a load cell placed in series with the external resistance, and moments were calculated using an inverse dynamics approach.

**Results:**

The range of motion and the joint loading was highly dependent on the exercises. The range of motion in the glenohumeral joint did not differ significantly between the two resistance types, whereas internal/external rotation moments were significantly higher with constant resistance than those with elastic resistance.

**Conclusions:**

Larger or lower moments can, therefore, be achieved through selection of the appropriate resistance type, while the range of motion can be altered through the selection of exercise type. Therefore, the loading motion patterns identified in this study can help to choose suitable shoulder exercises dependent on the training objective.

**Electronic supplementary material:**

The online version of this article (10.1186/s13102-018-0111-7) contains supplementary material, which is available to authorized users.

## Background

Following the back and knee, the shoulder is one of the most common sites for experiencing pain [1, 2], with shoulder injuries occurring in nearly all population groups [3–5]. The most common shoulder problem is related to injury to the rotator cuff [6, 7]. Furthermore, a deficit in rotator cuff strength, or an imbalanced muscular strength profile in the rotator cuff, is known to be associated with an increased risk of shoulder problems in various sports [8–11]. In fitness centres, the shoulder is the most common site of injury (24.4%), mainly due to overloading (45.6%) and incorrect exercise execution (21.1%) [5]. Therefore, rotator cuff muscles that are sufficiently strong and balanced are crucial for avoiding overload and averting shoulder pain/injury. Exercises that train the muscles of the rotator cuff are recommended for not only preventing injuries, but also for rehabilitating the shoulder after injury, as well as part of whole-body strength training programmes [12].

During training and in the fields of injury prevention and rehabilitation, exercises addressing the internal and external rotator muscles of the shoulder are generally either performed using constant resistance (CR) (e.g. pulley, dumbbell, or barbell) or elastic resistance (ER) (e.g. elastic straps or tubes). For the development of optimal training recommendations, the kinematic and kinetic nature of the specific exercises and training devices must be considered [13–15]. A previous analysis of the range of motion (RoM) and peak torque in the shoulder during external rotation exercises, performed with a cable pulley machine or with a variable resistance machine, found that the RoM and the angle at which the peak joint torque was achieved differed between the two training devices, even though the peak torque values did not differ significantly [16]. Furthermore, the resultant peak moments occurred at different angles when the exercises were performed with ER compared to when the same exercises were performed either without external load or with CR achieved through the use of dumbbells [17]. Moreover, the resultant peak moments during exercises performed with ER appeared to be smaller than those achieved when exercising with dumbbells [17]. Importantly, most previous studies examining kinetics or kinematics of the shoulder have investigated exercises other than internal or external rotation [17–20], and no studies have compared the three-dimensional (3D) shoulder kinetics and kinematics during internal and external rotation exercises performed with CR or with ER.

In biomechanical research, the 3D assessment of segmental motion based on skin markers is limited by soft tissue artefact [21], as well as marker visibility, marker cluster distribution, number of markers per segment, and positioning of the markers [13, 22, 23]. Since upper-body marker sets remain challenged by these issues, a suitable marker set for the shoulder is clearly required [20, 24–28]. Recent research has assessed the effect of different technical coordinate system definition on the three dimensional representation of the glenohumeral joint center [29]. It has been shown, that with a skin marker set it is possible to predict the position of the glenohumeral joint center [30]. Here, improved access to shoulder joint motion and loading conditions could open perspectives for a greater understanding of rotator cuff strengthening exercises and their applicability for targeted training and rehabilitation.

With the aim to establish exercise regimes that are able to provide high joint moments throughout a large rotational RoM for the rotator cuff muscles, the goal of the present study was therefore to examine upper extremity kinetics and kinematics in 3D for five internal and five external rotation exercises. All exercises are performed using two types of force application methods, including constant and elastic resistance. The second goal of the present study was to design a marker set for functional joint centre determination of the shoulder joints to be able to examine upper extremity kinetics and kinematics in 3D of the selected shoulder strength exercises. The range of motion between the two types of force application, constant and elastic resistance, is expected to show no difference. However, the maximal moments observed, as well as the angles of the maximal moments are assumed to differ between the selected exercises.

## Methods

### Participants

Six female and six male participants, all healthy and with experience in weight training (average age 24.9 ± 5.8 years; weight 68 ± 13 kg; height 1.76 ± 0.08 m) were recruited to participate in this study. An ethics application including method section, protocols, participant information and informed consent was submitted to and approved by the ethics committee of ETH Zurich, Switzerland (EK 2015-N-50). In addition, all participants provided written informed consent to participate before commencing testing. Inclusion criteria included participation in sport activities for at least three hours per week and participants were required to be physically fit and have basic experience in strength training. More details about inclusion and exclusion criteria are presented in Table 1.

**Table 1:** Selection criteria for participants according to the ethic application

Selection criteria:	· Participants do sport activities at least three hours per week. · Participants are physically fit (no current injuries) and have a RoM that is considered normal in the upper extremities. · Participants have basic personal experience in strength training. · Participants are between 18 and 45 years old.
Exclusion criteria:	· Past surgery on the upper extremities. · Current injury or illness. · Health problems. · Under medical treatment.

### Experimental approach

**Fig. 1:**
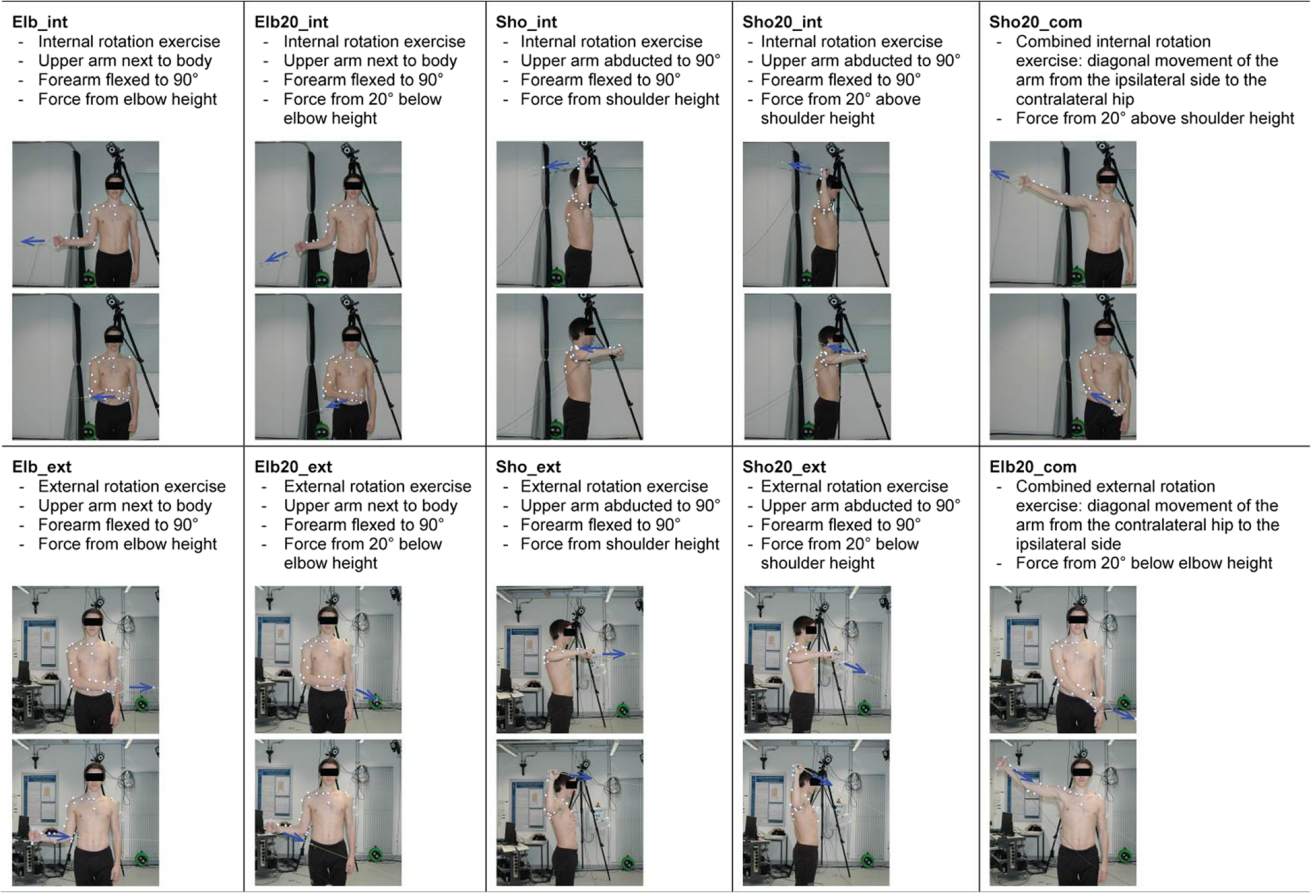
Internal and external rotation exercises performed by the participants using CR and ER. Arm positions and direction of the external force (blue arrow) differed for the various exercises. Exercises began with the concentric phase of the movement. Upper pictures correspond to the starting point of the concentric phase, lower pictures to the end point of the concentric phase

All participants performed eight repetitions of ten exercises using their dominant arm for coordination-based tasks, for which arm position and direction of the external force was varied. Five of the exercises are designed to train the internal rotator muscles of the shoulder (Elb_int, Elb20_int, Sho_int, Sho20_int, Sho20_com), and five the external rotator muscles (Elb_ext, Elb20_ext, Sho_ext, Sho20_ext, Elb20_com) (Fig. 1). Participants performed the exercises with a constant external load, or with an increasing external load due to its application via an elastic strap. Participants received standardized instructions for the execution of the exercises (see online Additional file 1). The order of execution was randomized and the maximum external load corresponded to 2% of the participant’s body mass (BM), as this was in the typical range of load used in rehabilitation training for the shoulder rotator muscles. When the exercises were performed with ER, the maximum load was always reached at the end of the concentric movement. All exercises started with the concentric phase first.

### Procedures

**Fig. 2:**
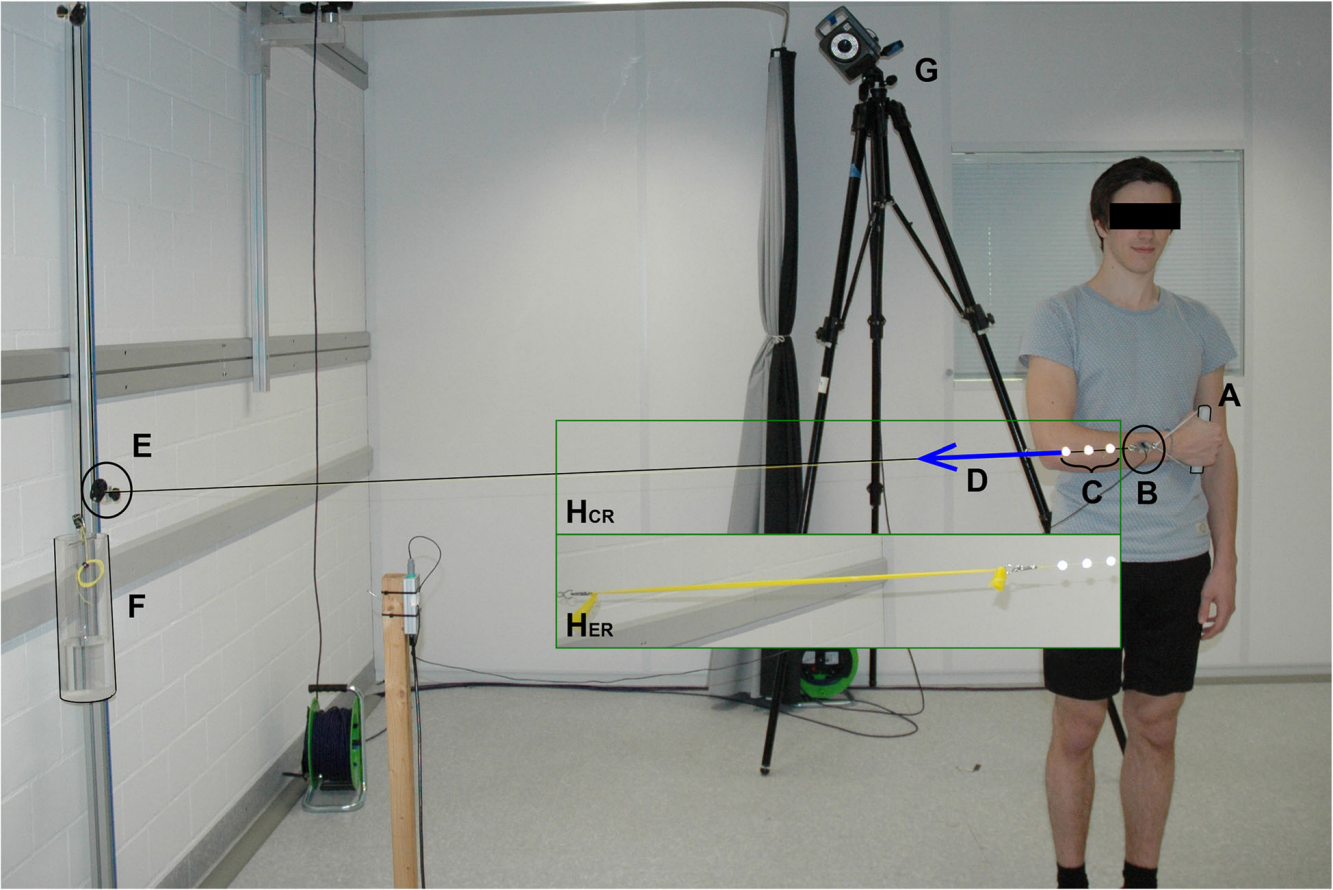
The measurement setup is shown. **a** handle, **b** load cell, **c** 3 markers for the determination of the direction of the external force, **d** external force, **e** height adjustable pulley, **f** water container for adjusting the weight, **g** one of the ten Vicon cameras, HCR) constant external resistance – could be replaced with HER) elastic external resistance

A pulley-like system was used to apply the CR, whereas a progressive resistance strap (yellow TheraBand®) was used for ER (Fig. 2). The external constant load was adjusted to 2% of the participant’s BM by adding or removing water mass. In order to prevent fatigue during the exercise the selected load was lower compared to previous work [17]. When not stretched, the resistance band was attached such that it had a functional length of 0.35 m. In order to modulate the elastic resistance behaviour to account for the required RoM during different exercises, the TheraBand® was cut lengthwise into thirds (for Elb20_com and Sho20_com) or in half (for all other exercises), thus reducing its stiffness by a factor of 3 or 2 respectively. The external elastic load was then adjusted by shortening or lengthening a non-elastic cord in order to obtain the same 2%BM load at the position of maximum TheraBand® length. With both training devices, the participants pulled on a handle that was fixed in series with either the constant or elastic resistance, and a load cell (KD24S, 50 N, Transmetra GmbH, Flurlingen, Switzerland) that measured the external load at a frequency of 1.2 kHz. Three-dimensional kinematics were evaluated using an optoelectronic motion capture system (Vicon, Oxford Metrics Group, UK) with 10 cameras (MX T160), which recorded the motion at a frequency of 100 Hz. No filtering or gap filling was applied to the marker trajectories during the post processing.

In order to dynamically capture the shoulder joint motion using a functionally determined glenohumeral joint centre (GHJC) in all anatomical planes and throughout all the rehabilitation exercises examined, a new marker set was developed based on the ISB recommended anatomical landmarks and supplemented further markers on the hand, forearm and upper arm [22, 28, 31]. The marker set consisted of 28 markers (Additional file 2: Figure S1), and aimed to ensure good marker visibility at all times, but also include a certain amount of redundancy in the marker cluster distributions [13, 22].

The location of the sternoclavicular joint centre (SCJC) was determined using a geometric approach, and was estimated to lie at a location 2/3 of the distance from the marker at the most cranial point of the sternum (STCR; Additional file 2: Figure S1) to the marker at the most medial point of the clavicula (RTSC). Importantly, the GHJC, the elbow joint centre (EJC), elbow joint axis (EJA), and wrist joint centre (WJC) were all functionally determined [22] based on 9 different basic motion tasks (BMT; a full description is presented in the Additional file 3: Table S1). All segmental coordinate systems were orthogonal and right-handed, and were determined based on the markers and on the estimated joint centres and axes (for definitions see Additional file 1).

Position and orientation of the segments were determined using a least-squares fit of the corresponding marker clusters [32]. Joint angles for every joint were calculated using the Joint Coordinate System (JCS) convention [33], for details please see (Additional file 2: Figure S1 and Additional file 1 “Definitions of the joint coordinate systems”). The RoMs were defined as the difference between the maximum and the minimum angles.

The external moments at the GHJC, EJC, and WJC were calculated using an inverse dynamics approach based on the positions of the segments, the measured external forces, and the gravitational force acting on the segments and handle, and were presented in components according to the non-orthogonal JCSs. Here, it is important to note that in a non-orthogonal system, the sum of the 3 components do not represent the absolute moment. The force data, measured by the load cell, were filtered using a 3rd order low-pass Butterworth filter and its direction was determined using three markers that were placed in series with the resistance cord and the load cell (Fig. 2). The mass and the centre of gravity for the upper arm, forearm, and hand, were determined according to Winter [34]. The mass of the handle was 0.047 kg and its centre of gravity was assumed to be the same as that for the hand. Moments and angles were resampled over time, and moments were additionally normalized to BM. All valid trials were analysed and no outliers were removed evaluation of the measured data. Mean peak values for all participants were calculated using the maximum and minimum values for each repetition. All calculations were performed using MATLAB (version R2014a, MathWorks, Natick, MA, USA).

### Statistical analysis

Defined parameters of interest were: RoM, maximum moment (M_max_), and the angle at which M_max_ occurred (α(M_max_)). All parameters for the GHJC were statistically evaluated around the internal/external (e_GH3_) and the adduction/abduction (e_GH2_) rotation axis. A statistical analysis of the SCJC was not performed. Comparisons between CR and ER, and between the different exercises, were performed, but with internal rotation exercises and external rotation exercises evaluated separately. For comparisons, a linear mixed model was utilized with the resistance type and exercise specified as fixed effects and participants specified as a random effect. Bonferroni-corrected post-hoc tests were conducted where appropriate using factor 6 according to the parameter examined (*p* = .00833). All statistical analyses were performed using SPSS software (version 22, IBM).

## Results

All parameters are displayed as mean ± standard deviation. Positive values correspond to internal rotation, adduction, or flexion angles or moments (Table 2, Table 3). The fixed and random effects coefficients of the linear mixed model are included in the Additional files section (Additional file 3: Tables S2-S4).

**Table 2:** Internal rotation exercises. Mean ± standard deviation of the internal/external rotation and adduction/abduction moments M_max_ [Nm/kg], RoMs [°], and angles at maximal moment α(M_max_) [°] in the GHJC are presented. Significant exercise differences (*p* < 0.05) are indicated in superscript form. *: represents values that are significantly different (p < 0.05) from those obtained with the other resistance type for the same exercise

			Elb_int		Elb20_int		Sho_int		Sho20_int		Sho20_com	
Internal/ external rotation in the GHJC	M_max_ [Nm/kg]	CR	−0.073 ± 0.006*	^Sho_int^	−0.066 ± 0.007*	^Sho_int^	− 0.086 ± 0.010*	^Elb_int^	−0.082 ± 0.006*	^Elb_int^	−0.032 ± 0.012*	^Elb_int^
				^Sho20_int^		^Sho20_int^		^Elb20_int^		^Elb20_int^		^Elb20_int^
				^Sho20_com^		^Sho20_com^		^Sho20_com^		^Sho20_com^		^Sho_int^
												^Sho20_int^
		ER	−0.041 ± 0.004*	^Sho20_com^	−0.039 ± 0.004*	^Sho20_int^	−0.047 ± 0.005*	^Sho20_com^	−0.041 ± 0.005*	^Elb20_int^	−0.014 ± 0.006*	^Elb_int^
						^Sho20_com^				^Sho20_com^		^Elb20_int^
												^Sho_int^
												^Sho20_int^
	RoM [°]	CR	92.6 ± 20.5	^Sho20_com^	90.4 ± 22.9	^Sho20_com^	79.2 ± 10.6	^Sho20_com^	79.7 ± 9.3	^Sho20_com^	110.0 ± 18.5	^Elb_int^
												^Elb20_int^
												^Sho_int^
												^Sho20_int^
		ER	96.1 ± 21.8	^Sho20_com^	91.1 ± 26.8	^Sho20_com^	81.1 ± 11.2	^Sho20_com^	81.5 ± 11.2	^Sho20_com^	112.3 ± 16.1	^Elb_int^
												^Elb20_int^
												^Sho_int^
												^Sho20_int^
	α(M_max_) [°]	CR	−11.6 ± 7.0*	^Sho_int^	−11.1 ± 7.1*	^Sho_int^	−68.3 ± 8.2	^Elb_int^	−65.2 ± 6.5	^Elb_int^	−9.3 ± 11.7*	^Sho_int^
				^Sho20_int^		^Sho20_int^		^Elb20_int^		^Elb20_int^		^Sho20_int^
								^Sho20_com^		^Sho20_com^		
		ER	7.9 ± 5.8*	^Sho_int^	6.3 ± 5.7*	^Sho_int^	−69.0 ± 7.3	^Elb_int^	−67.6 ± 8.4	^Elb_int^	26.5 ± 10.2*	^Elb_int^
				^Sho20_int^		^Sho20_int^		^Elb20_int^		^Elb20_int^		^Elb20_int^
				^Sho20_com^		^Sho20_com^		^Sho20_com^		^Sho20_com^		^Sho_int^
												^Sho20_int^
Adduction/ abduction in the GHJC	M_max_ [Nm/kg]	CR	−0.067 ± 0.019	^Sho_int^	−0.083 ± 0.022	^Sho_int^	0.071 ± 0.014	^Elb_int^	0.067 ± 0.010	^Elb_int^	−0.082 ± 0.013	^Sho_int^
				^Sho20_int^		^Sho20_int^		^Elb20_int^		^Elb20_int^		^Sho20_int^
								^Sho20_com^		^Sho20_com^		
		ER	−0.073 ± 0.019	^Sho_int^	−0.086 ± 0.022	^Sho_int^	0.072 ± 0.014	^Elb_int^	0.067 ± 0.011	^Elb_int^	−0.084 ± 0.013	^Sho_int^
				^Sho20_int^		^Sho20_int^		^Elb20_int^		^Elb20_int^		^Sho20_int^
								^Sho20_com^		^Sho20_com^		
	RoM [°]	CR	11.5 ± 6.5	^Sho20_com^	11.1 ± 6.1	^Sho20_com^	10.2 ± 4.1	^Sho20_com^	9.1 ± 4.4	^Sho20_com^	87.9 ± 16.1	^Elb_int^
												^Elb20_int^
												^Sho_int^
												^Sho20_int^
		ER	14.4 ± 7.8	^Sho20_com^	12.3 ± 6.9	^Sho20_com^	10.2 ± 6.1	^Sho20_com^	9.6 ± 3.4	^Sho20_com^	87.0 ± 16.2	^Elb_int^
												^Elb20_int^
												^Sho_int^
												^Sho20_int^
	α(M_max_) [°]	CR	−3.6 ± 8.4	^Sho_int^	−3.9 ± 7.4	^Sho_int^	− 61.4 ± 10.0	^Elb_int^	−63.0 ± 9.2	^Elb_int^	10.9 ± 7.7	^Elb_int^
				^Sho20_int^		^Sho20_int^		^Elb20_int^		^Elb20_int^		^Elb20_int^
				^Sho20_com^		^Sho20_com^		^Sho20_com^		^Sho20_com^		^Sho_int^
												^Sho20_int^
		ER	−0.4 ± 7.4	^Sho_int^	−1.8 ± 7.5	^Sho_int^	−61.5 ± 10.3	^Elb_int^	−62.1 ± 12.2	^Elb_int^	10.8 ± 7.6	^Elb_int^
				^Sho20_int^		^Sho20_int^		^Elb20_int^		^Elb20_int^		^Elb20_int^
				^Sho20_com^		^Sho20_com^		^Sho20_com^		^Sho20_com^		^Sho_int^
												^Sho20_int^

**Table 3:** External rotation exercises. Mean ± standard deviation of the internal/external rotation and adduction/abduction moments M_max_ [Nm/kg], RoMs [°], and angles at maximal moment α(M_max_) [°] in the GHJC are presented. Significant exercise differences (p < 0.05) are indicated in superscript form. *: represents values that are significantly different (p < 0.05) from those obtained with the other resistance type for the same exercise

			Elb_ext		Elb20_ext		Sho_ext		Sho20_ext		Elb20_com	
Internal/ external rotation in the GHJC	M_max_ [Nm/kg]	CR	0.085 ± 0.005*	^Sho20_ext^	0.088 ± 0.006*	^Sho20_ext^	0.088 ± 0.006*	^Sho20_ext^	0.103 ± 0.004*	^Elb_ext^	0.070 ± 0.020*	^Elb_ext^
				^Elb20_com^		^Elb20_com^		^Elb20_com^		^Elb20_ext^		^Elb20_ext^
										^Sho_ext^		^Sho_ext^
										^Elb20_com^		^Sho20_ext^
		ER	0.057 ± 0.005*	^Sho_ext^	0.060 ± 0.006*	^Sho20_ext^	0.069 ± 0.006*	^Elb_ext^	0.075 ± 0.007*	^Elb_ext^	0.046 ± 0.013*	^Elb_ext^
				^Sho20_ext^		^Elb20_com^		^Elb20_com^		^Elb20_ext^		^Elb20_ext^
				^Elb20_com^						^Elb20_com^		^Sho_ext^
												^Sho20_ext^
	RoM [°]	CR	89.8 ± 22.7	^Sho_ext^	85.5 ± 20.7	^Sho_ext^	69.1 ± 9.3	^Elb_ext^	76.5 ± 9.2	^Elb_ext^	113.6 ± 17.5	^Elb_ext^
				^Sho20_ext^		^Sho20_ext^		^Elb20_ext^		^Elb20_ext^		^Elb20_ext^
				^Elb20_com^		^Elb20_com^		^Elb20_com^		^Elb20_com^		^Sho_ext^
												^Sho20_ext^
		ER	96.1 ± 19.8	^Sho_ext^	92.9 ± 21.8	^Sho_ext^	68.9 ± 10.8	^Elb_ext^	75.8 ± 11.1	^Elb_ext^	112.5 ± 15.4	^Elb_ext^
				^Sho20_ext^		^Sho20_ext^		^Elb20_ext^		^Elb20_ext^		^Elb20_ext^
				^Elb20_com^		^Elb20_com^		^Elb20_com^		^Elb20_com^		^Sho_ext^
												^Sho20_ext^
	α(M_max_) [°]	CR	0.2 ± 7.7*	^Sho_ext^	−1.4 ± 5.7*	^Sho_ext^	−34.9 ± 5.9*	^Elb_ext^	−22.1 ± 5.9*	^Elb_ext^	16.0 ± 8.4*	^Elb_ext^
				^Sho20_ext^		^Sho20_ext^		^Elb20_ext^		^Elb20_ext^		^Elb20_ext^
				^Elb20_com^		^Elb20_com^		^Sho20_ext^		^Sho_ext^		^Sho_ext^
								^Elb20_com^		^Elb20_com^		^Sho20_ext^
		ER	−13.1 ± 5.9*	^Sho_ext^	−13.5 ± 7.3*	^Sho_ext^	−43.0 ± 6.8*	^Elb_ext^	−30.7 ± 8.6*	^Elb_ext^	−6.8 ± 7.3*	^Sho_ext^
				^Sho20_ext^		^Sho20_ext^		^Elb20_ext^		^Elb20_ext^		^Sho20_ext^
								^Sho20_ext^		^Sho_ext^		
								^Elb20_com^		^Elb20_com^		
Adduction/ abduction in the GHJC	M_max_ [Nm/kg]	CR	0.077 ± 0.012	^Sho_ext^	0.090 ± 0.018	^Sho20_ext^	0.106 ± 0.014	^Elb_ext^	0.123 ± 0.017	^Elb_ext^	0.136 ± 0.033	Elb-ext
				^Sho20_ext^		^Elb20_com^		^Elb20_com^		^Elb20_ext^		^Elb20_ext^
				^Elb20_com^								^Sho_ext^
		ER	0.084 ± 0.017	^Sho20_ext^	0.096 ± 0.011	^Sho20_ext^	0.103 ± 0.013	^Elb20_com^	0.124 ± 0.013	^Elb_ext^	0.141 ± 0.029	^Elb_ext^
				^Elb20_com^		^Elb20_com^				^Elb20_ext^		^Elb20_ext^
												^Sho_ext^
	RoM [°]	CR	10.3 ± 5.9	^Elb20_com^	8.6 ± 4.1	^Elb20_com^	14.2 ± 3.1	^Elb20_com^	12.5 ± 4.2	^Elb20_com^	95.7 ± 19.1	^Elb_ext^
												^Elb20_ext^
												^Sho_ext^
												^Sho20_ext^
		ER	11.0 ± 5.0	^Elb20_com^	10.3 ± 4.5	^Elb20_com^	13.6 ± 3.9	^Elb20_com^	13.2 ± 5.5	^Elb20_com^	90.4 ± 16.7	^Elb_ext^
												^Elb20_ext^
												^Sho_ext^
												^Sho20_ext^
	α(M_max_) [°]	CR	−13.7 ± 4.3	^Sho_ext^	−14.0 ± 4.0	^Sho_ext^	−55.2 ± 7.8	^Elb_ext^	−57.9 ± 7.8	^Elb_ext^	−70.9 ± 16.8	^Elb_ext^
				^Sho20_ext^		^Sho20_ext^		^Elb20_ext^		^Elb20_ext^		^Elb20_ext^
				^Elb20_com^		^Elb20_com^		^Elb20_com^		^Elb20_com^		^Sho_ext^
												^Sho20_ext^
		ER	−11.7 ± 5.4	^Sho_ext^	−12.7 ± 4.6	^Sho_ext^	−53.5 ± 8.1	^Elb_ext^	−53.6 ± 10.2	^Elb_ext^	−71.0 ± 13.2	^Elb_ext^
				^Sho20_ext^		^Sho20_ext^		^Elb20_ext^		^Elb20_ext^		^Elb20_ext^
				^Elb20_com^		^Elb20_com^		^Elb20_com^		^Elb20_com^		^Sho_ext^
												^Sho20_ext^

### Kinematics

The RoM in the GHJC did not differ significantly between CR and ER for any exercise. During the Sho20_com and Elb20_com exercises, the RoMs for internal/external rotation and adduction/abduction were significantly higher than those for all other internal and external rotation exercises (Table 2, Table 3).

No statistical analysis was performed for the RoMs in the SCJC, but maximal RoMs were achieved during the Sho20_com exercise performed with ER and the Elb20_com exercise performed with CR, which reached up to 16.9 ± 3.5° for internal/external rotation, 22.7 ± 4.7° for adduction/abduction, and 13.3 ± 5.0° for flexion/extension.

### Kinetics

**Fig. 3:**
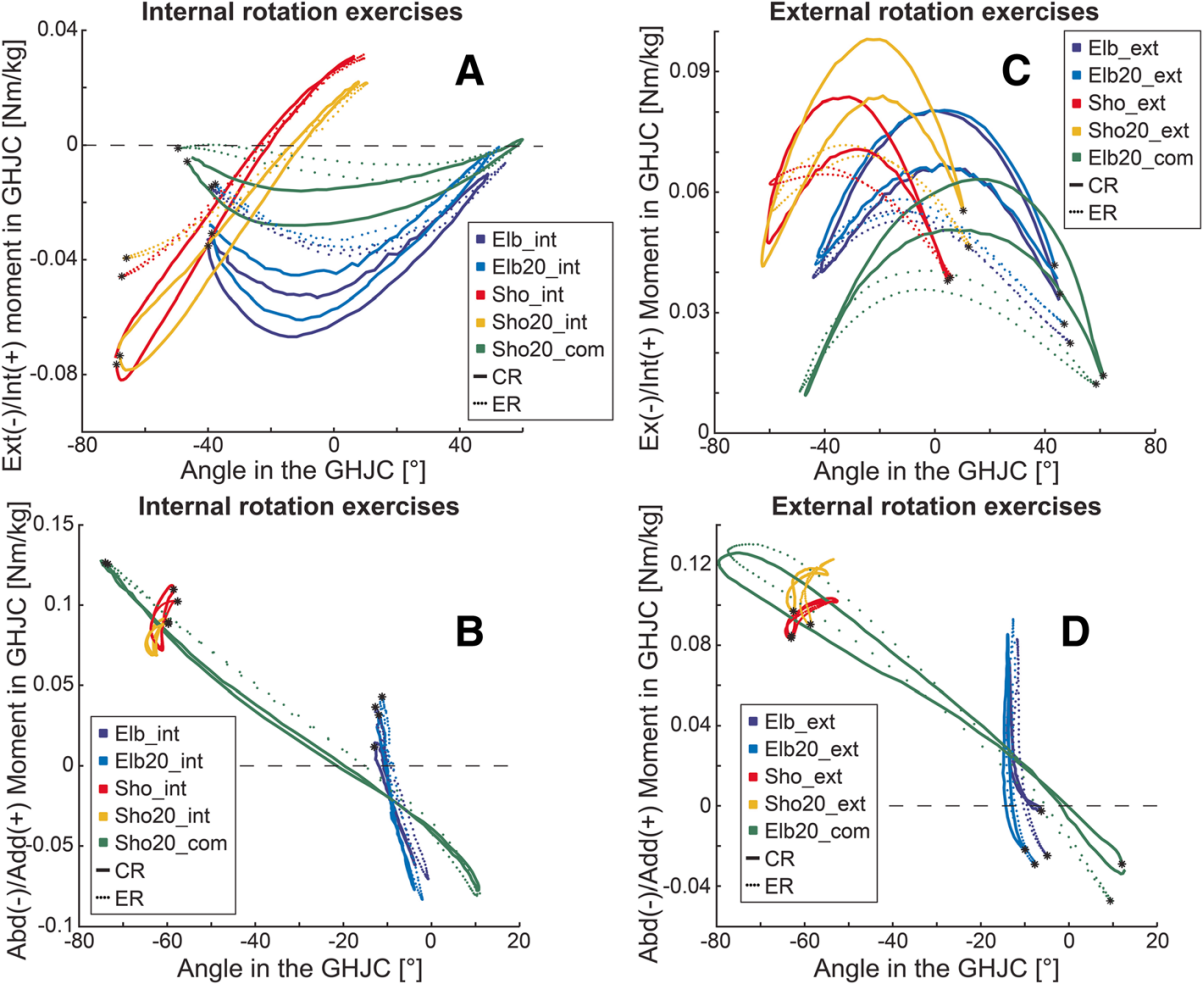
Joint moments and angles in the GHJC: **a** Normalized internal/external rotation moments [Nm/kg] as a function of the internal/external rotation angles [°] are shown for internal rotation exercises of the shoulder. **b** Normalized adduction/abduction moments [Nm/kg] as a function of the adduction/abduction angles [°] are shown for internal rotation exercises of the shoulder. **c** Normalized internal/external rotation moments [Nm/kg] as a function of the internal/external rotation angles [°] are shown for external rotation exercises of the shoulder. **d** Normalized adduction/abduction moments [Nm/kg] as a function of the adduction/abduction angles [°] are shown for external rotation exercises of the shoulder. The joint angles are defined the follows: int(+)/ext.(−) rotation und add(+)/abd(−). *: Starting point of the concentric phase of the movement

In the GHJC, the maximum external moment during internal rotation exercises, and the maximum internal moment, M_max_, during external rotation exercises were significantly higher when the exercise was performed with CR than when performed with ER (Tables 2 and 3). The highest external moment was obtained while performing the Sho_int and Sho20_int exercises with CR, whereas the highest internal moment was achieved during the Sho20_ext exercise, also performed with CR (Fig. 3a). Sho20_com and Elb20_com exercises led to the lowest internal and external maximal moment for both resistance types. In the GHJC, the adduction/abduction M_max_ was not significantly different between the CR and ER configurations (Table 2 and 3). It is important to note that the joint moments for Sho_int and Sho20_int exercises changed from an external rotation moment to an internal rotation moment and back again during the exercise execution (Fig. 3a), but this phenomenon did not occur for any of the moments in external rotation (Fig. 3c).

During the Sho_int and Sho20_int exercises, adduction moments occurred throughout the whole repetition, while the other internal rotation exercises exhibited moments that switched from an adduction to an abduction, and back to an adduction moment again (Fig. 3b). Similarly, the moments for Elb_ext, Elb20_ext, and Elb20_com exercises changed from an abduction moment to an adduction moment and back, while an adduction moment was obtained throughout the whole exercise execution for Sho_ext and Sho20_ext (Fig. 3d).

### Exercise comparisons

The internal/external rotation angle at which the maximal moment M_max_ occurred, α(M_max_), was significantly different between the two resistance types for all exercises except for Sho_int (*p* = 0.396) and Sho20_int (*p* = 0.794) (Table 2 and 3). The maximum external moment, M_max_, in Sho20_com, Elb_int, and Elb20_int exercises occurred during the first half of the concentric phase of the movement when performed with CR, but occurred later in the movement when performed with ER (Fig. 3a). Furthermore, in the external rotation exercises, the maximum internal moment was achieved earlier in the concentric phase with CR than with ER for all exercises (Fig. 3c). The adduction/abduction angle, α(M_max_), was not significantly different between CR and ER (internal and external rotational exercises *p* = 0.935 and *p* = 0.919, respectively) (Table 2 and 3). No differences in the examined parameters were observed between Elb_int and Elb20_int, between Sho_int and Sho20_int, or between Elb_ext and Elb20_ext exercises. In contrast, Sho_ext and Sho20_ext exercises differed in internal M_max_ with CR, and in the internal α(M_max_) with both CR and ER (Table 3).

## Discussion

Internal and external rotation exercises for the shoulder are often performed during both rehabilitation and training sessions. The biomechanical assessment of these exercises is important for the establishment of evidence based guidelines; however, only sparse research has been performed in this area. To the authors’ knowledge, the present study is the first to examine the RoMs and corresponding joint moments in the upper extremities during internal and external rotation exercises of the shoulder, performed using CR and ER. Due to the lack of previous investigations, comparison of the current results with previous results is difficult; thus, further studies are needed to verify the outcomes of the present study.

For both CR and ER, the external loading at the end of the concentric movement phase corresponded to approximately 2% of the participant’s BM. Therefore, as expected in the first part of our hypothesis, the RoM did not differ significantly between the two resistance types. Importantly, however, the maximum internal/external rotation moment, as significantly higher with CR than with ER. The second hypothesis, assuming a difference of M_max_ and α(M_max_) for the different exercises has been confirmed. Furthermore, the joint angle at which the maximal moment occurred, α(M_max_), was generally achieved earlier in the concentric movement phase with CR than with ER. Similar to our results, de Toledo, Ribeiro [17] demonstrated that M_max_ and α(M_max_) were statistically different when performing exercises with ER compared with CR using dumbbells, even if the reported moments appear to be slightly higher than those presented in the current study. The different moments are likely to have occurred due to different movement patterns in the two studies. Changes in the joint moments between ER and CR indicate that the resultant M_max_ should be taken into account when selecting the appropriate resistance type. This new knowledge of the interaction between joint angles and corresponding moments enhances the previous understanding of muscle usage with different resistance types [35], where EMG based-muscle activity suggested that either dumbbells or elastic tubing could be equally chosen by therapists in clinical practice. In our study, various CRs resulted in higher internal and external joint moments in the GHJC compared with exercises using ER, and as a result, the corresponding muscles will be trained more when using CR.

The largest RoMs in the GHJC were observed during the Sho20_com and Elb20_com exercises. In addition, these exercises led to the lowest maximum external and internal joint moment. In order to achieve a high training effect, strengthening exercises should be performed over the entire RoM [36]. Therefore, Sho20_com and Elb20_com exercises might be valuable for patients undergoing shoulder internal/external rotation rehabilitation training, who should not overload the body structures in question, but still need to perform movements over large RoMs. Working with a CR (e.g. pulley system) at loads higher than 50% of the one repetition maximum has been shown to significantly decrease the RoMs in the GHJC compared to that with lower loads [16]. Hence, in order to train over large RoMs with suitable loads, working with a variable resistance machine should be considered in order to maintain the RoM (Peltonen et al., 2012). As a result, it would seem to be important for therapists to carefully increase the load with progressive rehabilitation so that the patients are still able to maintain a large RoM during the exercises.

Exercises that were performed with the external force running parallel to the floor yielded almost no significant parameter differences from exercises performed with an inclination of 20°. Only the Sho_ext and Sho20_ext exercises exhibited different values for M_max_ (only within CR) and for α(M_max_). These results indicate that, for the most part, the kinetics/kinematics of the shoulder exercises examined do not depend on whether the external force runs parallel to the floor or with a slight inclination angle. However, if an external rotation exercise is performed with the arm abducted to 90°, the direction of the external force must be chosen carefully. Note that the external force was not inclined upwards and downwards in the present study, but only in one direction depending on the exercise (Fig. 1). Further research is clearly required to investigate whether an additional rotation of the arm or different angle of the load application is required in these exercises in order to enhance the desired muscular loading over a large RoM.

Against expectations, all internal rotation exercises led to adduction moments, either during the whole movement cycle (Sho_int and Sho20_int) or during part of the movement cycle (Elb_int, Elb20_int, and Sho20_com). Importantly, the adduction/abduction moments in the Elb_ext, Elb20_ext, and Elb20_com exercises, and the internal/external rotation moments in the Sho_int and Sho20_int exercises changed signs over the movement cycle. This effect was considered to occur because the weight of the arm inducing a moment in the GHJC was too high to be fully compensated by the small external forces (approx. 2%BM). Exercises should therefore be chosen carefully, depending on the training goal: e.g. if the abduction muscles need to be strengthened, internal rotation exercises should be chosen with caution as they lead to both agonistic and antagonistic loading in the GHJC and therefore train adduction muscles rather than abduction muscles over at least part of the movement.

Studies that examined kinematics of the shoulder have often only investigated the motion of the humerus or scapula relative to the torso [18, 37]. However, as the shoulder complex consists of three joints that all contribute to the total motion of the arm, it is clear than more than one joint should be investigated simultaneously. However, tracking the movement of the scapula as well as the clavicula is difficult because of skin motion artefacts [38]. As the upper extremities are linked to the torso via the sternoclavicular joint, one possible approach to investigate upper extremity kinematics is to model the shoulder girdle as a single segment and examine the relative motion between the shoulder girdle and the humerus, as well as between the shoulder girdle and the torso. In the present study, the measured RoMs in the SCJC demonstrated substantial movement of the shoulder girdle relative to the torso. In our study, the motion of the humerus relative to the torso can also be examined by adding the angles obtained in the GHJC and the SCJC, allowing comparisons with studies using the previously mentioned approach. However, it should be mentioned that our model did not reflect motion/rotation of the scapula or the clavicula, but of the shoulder girdle as a whole.

For the GHJC, the flexion/extension axis was defined to be the floating axis (FL), as this was the axis of least importance for the examined movements. With this approach, however, it is possible that gimbal lock occurs, and that the angles cannot be defined over the whole flexion/extension RoMs. Nevertheless, this approach has been chosen for the description of the angles in order to ensure direct clinical interpretation. Therefore, interpretation of the flexion/extension angles in the GHJC must be treated with caution, and if other movements in the shoulder joint are to be examined, the JCS may need to be defined differently. Another limitation of this study is, that the here used marker set has not been directly validated with a gold standard such as MRI.

## Conclusions

Depending on the desired RoMs, different exercises can be chosen. The largest RoMs in both internal/external rotation and adduction/abduction direction can be achieved performing the Sho20_com or Elb20_com exercises. However, the higher complexity of the combined movements needs more cautious interpretation due to gimbal lock, limited clinical interpretability and dependency of resulting joint motion on the choice of mathematical convention for joint angular description. In contrast, M_max_ and α(M_max_) can be altered by the choice of the resistance type. The maximum internal/external moment, M_max_, was larger in exercises performed with CR than in those performed with ER, while α(M_max_) occurred earlier in the concentric movement with CR than with ER. To produce larger moments throughout the full RoM using the same maximum external force, exercises should therefore be performed using CR. Exercises found to change moment sign during the movement repetition should be selected with care, depending on the training goals, in order to focus on the intended target muscles and not their antagonists. In particular, Sho_int and Sho20_int might not be appropriate for rehabilitation, as they result in adduction moments rather than the expected abduction moments, and to internal rotation moments over part of the movement in addition to the expected external rotation moments. Exercises with a large moment over a large RoM are considered to be the most efficient exercises. If, for instance, internal rotation muscles should be trained, Elb_int or Elb20_int would be reasonable choices, as these exercises show a good trade-off between the examined parameters. Furthermore, whether the external force runs parallel to the ground or with an inclination angle of up to 20° downwards does not appear to have a large influence and can be selected as desired.

## Additional files

Additional file 1: Description of the coordinate systems. Definition of the segmental coordinate system and the joint coordinate centers. (PDF 161 kb)

Additional file 2: The marker set for a right-handed participant is shown with the joint coordinate systems. A total of 28 markers were used to model the following segments: torso, shoulder girdle, upper arm, forearm, and hand. The contribution of the shoulder girdle to arm motion should not be neglected; therefore, the scapula and clavicula were modelled as a single segment (shoulder girdle). Hand markers had a diameter of 9 mm while the remaining markers had a diameter of 14 mm. (TIF 4823 kb)

Additional file 3: **Table S1.** Basic motion tasks. **Tables S2.** and **S3.** Fixed effects coefficients and standard error. **Table S4.** Random effects coefficients and standard error. (PDF 224 kb)

## Abbreviations

3D: three dimensional
BM: body mass
BMT: basic motion tasks
CR: constant resistance
EJA: elbow joint axis
EJC: elbow joint centre
EMG: electro myography
ER: elastic resistance
GHJC: Glenohumeral joint center
Mmax: maximal joint moment
RoM: Range of Motion
SCJC: sterniclavicular joint centre
WJC: wrist joint centre
α(Mmax): joint angle at the maximal joint moment
