# Supplementary material for: Comparison of the kinematics and kinetics of shoulder exercises performed with constant and elastic resistance
Source: BMC Sports Sci Med Rehabil. 2018 Nov 28;10:22. doi: 10.1186/s13102-018-0111-7 (PMC6262970; doi:10.1186/s13102-018-0111-7)
Supplement: Supplementary file 1 — Description of the coordinate systems. Definition of the segmental coordinate system and the joint coordinate centers. (PDF 161 kb) [file 13102_2018_111_MOESM1_ESM.pdf]

Supplementary Material 1: Definition of the segmental coordinate system and the joint coordinate centers.

The segmental coordinate systems were defined as follows:
1) Torso coordinate system:
$\overrightarrow{eto1}$ (mediolateral axis) is perpendicular to the plane formed by the four torso markers.
$\overrightarrow{eto3}$ (vertical axis) is perpendicular to $\overrightarrow{eto1}$ and lies in the plane formed by the connecting line
between the middle points of the markers STCA/SPT8 and STCR/SPC7 and $\overrightarrow{eto1}$.
$\overrightarrow{eto2}$ (anteroposterior axis) is perpendicular to $\overrightarrow{eto1}$ and $\overrightarrow{eto3}$.

2) Shoulder girdle coordinate system:
$\overrightarrow{esg1}$ (mediolateral axis) is the connecting line between the left and the right acromion
markers.
$\overrightarrow{esg3}$ (vertical axis) is perpendicular to $\overrightarrow{esg1}$ and lies in the plane formed by the connecting line between the GHJC calculated as in Rab et al [16] and the acromion marker and $\overrightarrow{esg1}$.
$\overrightarrow{esg2}$ (anteroposterior axis) is perpendicular to $\overrightarrow{esg1}$ and $\overrightarrow{esg3}$.

3) Upper arm coordinate system:
$\overrightarrow{eua1}$ (mediolateral axis) corresponds to the functionally estimated EJA.
$\overrightarrow{eua3}$ (vertical axis) is perpendicular to eua1 and lies in the plane formed by the GHJC and $\overrightarrow{eua1}$.
eua2 (anteroposterior axis) is perpendicular to $\overrightarrow{eua1}$ and $\overrightarrow{eua3}$.

4) Forearm coordinate system:
$\overrightarrow{efa3}$ (vertical axis) is the connecting line between the WJC and the EJC.
$\overrightarrow{efa1}$ (mediolateral axis) is perpendicular to $\overrightarrow{efa3}$ and lies in the plane formed by the markers WRA and WRB and the EJC.
$\overrightarrow{efa2}$ (anteroposterior axis) is perpendicular to $\overrightarrow{efa1}$ and $\overrightarrow{efa3}$.

5) Hand coordinate system:
$\overrightarrow{eha2}$ (anteroposterior axis) is perpendicular to the plane formed by the four hand markers.

$\overrightarrow{eha3}$ (vertical axis) is perpendicular to $\overrightarrow{eha2}$ and lies in the plane formed by the connecting line between the middle points of the markers DM2/DM5 and CM2/CM5 and $\overrightarrow{eha2}$.
$\overrightarrow{eha1}$ (mediolateral axis) is perpendicular to $\overrightarrow{eha2}$ and $\overrightarrow{eha3}$.

Joint coordinate systems were defined the follows:
1) Sternoclavicular joint coordinate system:
$\overrightarrow{eSC1}$ (flexion/extension axis) is fixed at the proximal segment (torso) and corresponds to $\overrightarrow{eto1}$.
$\overrightarrow{eSC3}$ (internal/external rotation axis) is fixed at the distal segment (shoulder girdle) and corresponds to $\overrightarrow{esg3}$.
$\overrightarrow{eSC2}$ = floating axis (adduction/abduction axis) is perpendicular to $\overrightarrow{eSC1}$ and $\overrightarrow{eSC3}$.

2) Glenohumeral joint coordinate system:
$\overrightarrow{eGH2}$ (adduction/abduction axis) is fixed at the proximal segment (shoulder girdle) and corresponds to $\overrightarrow{esg2}$.
$\overrightarrow{eGH3}$ (internal/external rotation axis) is fixed at the distal segment (upper arm) and corresponds to $\overrightarrow{eua3}$.
$\overrightarrow{eGH1}$ = floating axis (flexion/extension axis) is perpendicular to $\overrightarrow{eGH2}$ and $\overrightarrow{eGH3}$

3) Elbow joint coordinate system:
$\overrightarrow{eEL1}$ (flexion/extension axis) is fixed at the proximal segment (upper arm) and corresponds to $\overrightarrow{eua1}$.
$\overrightarrow{eEL3}$ (internal/external rotation axis) is fixed at the distal segment (forearm) and corresponds to $\overrightarrow{efa3}$.
$\overrightarrow{eEL2}$ = floating axis (adduction/abduction axis) is perpendicular to $\overrightarrow{eEL1}$ and $\overrightarrow{eEL3}$.

4) Wrist joint coordinate system:
$\overrightarrow{eWR1}$ (flexion/extension axis) is fixed at the proximal segment (forearm) and corresponds to $\overrightarrow{efa1}$.
$\overrightarrow{eWR3}$ (internal/external rotation axis) is fixed at the distal segment (hand) and corresponds to $\overrightarrow{eha3}$.
$\overrightarrow{eWR2}$ = floating axis (adduction/abduction axis) is perpendicular to $\overrightarrow{eWR1}$ and $\overrightarrow{eWR3}$.
