# Supplementary figures and images for: Comparison of the kinematics and kinetics of shoulder exercises performed with constant and elastic resistance
Source: BMC Sports Sci Med Rehabil. 2018 Nov 28;10:22. doi: 10.1186/s13102-018-0111-7 (PMC6262970; doi:10.1186/s13102-018-0111-7)

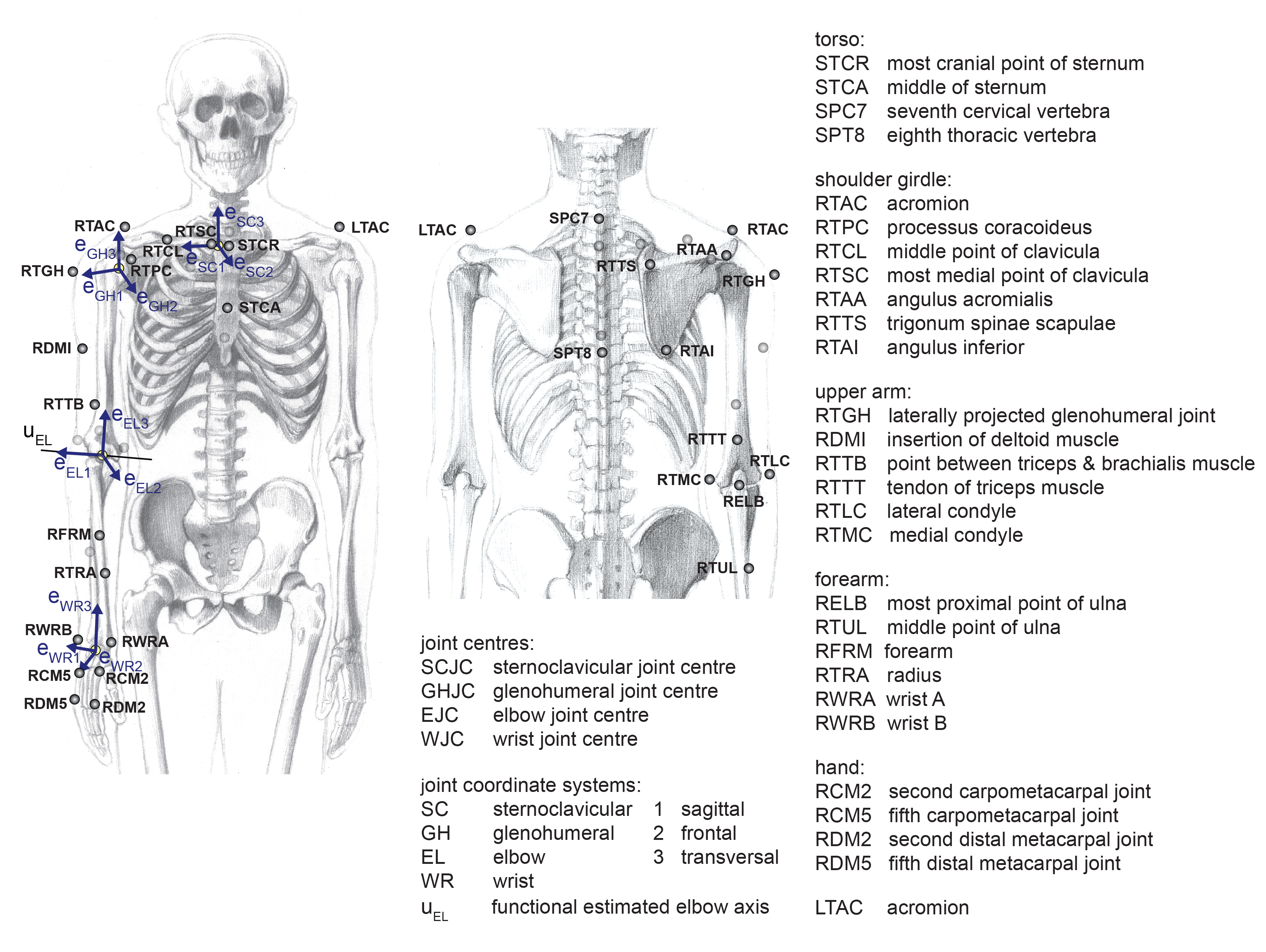

Supplement: Supplementary file 2 — The marker set for a right-handed participant is shown with the joint coordinate systems. A total of 28 markers were used to model the following segments: torso, shoulder girdle, upper arm, forearm, and hand. The contribution of the shoulder girdle to arm motion should not be neglected; therefore, the scapula and clavicula were modelled as a single segment (shoulder girdle). Hand markers had a diameter of 9 mm while the remaining markers had a diameter of 14 mm. (TIF 4823 kb) [file 13102_2018_111_MOESM2_ESM.tif]
