# Supplementary material for: Comparison of the kinematics and kinetics of shoulder exercises performed with constant and elastic resistance
Source: BMC Sports Sci Med Rehabil. 2018 Nov 28;10:22. doi: 10.1186/s13102-018-0111-7 (PMC6262970; doi:10.1186/s13102-018-0111-7)
Supplement: Supplementary file 3 — Table S1. Basic motion tasks. Tables S2. and S3. Fixed effects coefficients and standard error. Table S4. Random effects coefficients and standard error. (PDF 224 kb) [file 13102_2018_111_MOESM3_ESM.pdf]

Table S1: Basic motion tasks (BMT) for the functional estimation of the GHJC, EJC, EJA, and WJC

Shoulder BMT1	Flexion/extension movement in the shoulder in the sagittal plane while keeping the arm straight (3 repetitions)
Shoulder BMT2	Abduction/adduction movement in the shoulder in the frontal plane while keeping the arm straight (3 repetitions)
Shoulder BMT3	Horizontal flexion/extension movement in the shoulder in the transversal plane while keeping the arm straight (3 repetitions)
Shoulder BMT4	Circumduction movement in the shoulder while keeping the arm straight (3 repetitions)
Elbow BMT1	Flexion/extension movement in the elbow with a flexion angle of 90° in the shoulder and the thumb pointing upwards (3 repetitions)
Elbow BMT2	Pronation/supination movement in the elbow with a flexion angle of 90° in the shoulder (3 repetitions)
Wrist BMT1	Dorsal extension/palmar flexion movement in the wrist with a flexion angle of 90° in the shoulder (3 repetitions)
Wrist BMT2	Radial/ulnar deviation movement in the wrist with a flexion angle of 90° in the shoulder (3 repetitions)
Wrist BMT3	Circumduction movement in the wrist with a flexion angle of 90° in the shoulder (3 repetitions)

TableS 2: Fixed effects coefficients (β) and standard error (SE) of the mixed linear model for the internal rotation exercise

		**Intercept**		**CR**		**Elb_int**		**Elb20_int**		**Sho20_int**		**Sho20_com**		**[CR]*[Elb_int]**		**[CR]*[Elb20_int]**		**[CR]*[Sho20_int]**		**[CR]*[Sho20_com]**	
		β	SE(β)	β	SE(β)	β	SE(β)	β	SE(β)	β	SE(β)	β	SE(β)	β	SE(β)	β	SE(β)	β	SE(β)	β	SE(β)
**Internal/ external rotation in the GHJC**	**M_{max} [Nm/kg]**	-0.047	0.002	-0.039	0.002	0.006	0.002	0.009	0.002	0.007	0.002	0.033	0.002	0.007	0.003	0.012	0.003	-0.003	0.003	0.022	0.003
	**RoM [°]**	81.094	5.152	-1.880	4.464	15.017	4.464	9.972	4.464	0.353	4.464	31.234	4.464	-1.670	6.314	1.179	6.314	0.156	6.314	-0.482	6.314
	**$\alpha(M_{max})$ [°]**	-69.025	2.309	0.749	2.865	76.964	2.865	75.290	2.865	1.410	2.865	95.564	2.865	-20.322	4.051	-18.072	4.051	1.695	4.051	-36.612	4.051
**Adduction/ abduction in the GHJC**	**M_{max} [Nm/kg]**	0.072	0.005	-0.001	0.006	-0.145	0.006	-0.158	0.006	-0.005	0.006	-0.156	0.006	0.008	0.008	0.004	0.008	0.002	0.008	0.003	0.008
	**RoM [°]**	10.198	2.574	0.038	3.139	4.148	3.139	2.088	3.139	-0.582	3.139	76.826	3.139	-2.840	4.439	-1.196	4.439	-0.559	4.439	0.832	4.439
	**$\alpha(M_{max})$ [°]**	-61.466	2.562	0.071	3.067	61.034	3.067	59.689	3.067	-0.611	3.067	72.296	3.067	-3.201	4.338	-2.140	4.338	-0.981	4.338	-0.026	4.338

Table S3: Fixed effects coefficients (β) and standard error (SE) of the mixed linear model for the external rotation exercise

		Intercept		CR		Elb_ext		Elb20_ext		Sho20_ext		Elb20_com		[CR]*[Elb_ext]		[CR]*[Elb20_ext]		[CR]*[Sho20_ext]		[CR]*[Elb20_com]	
		β	SE(β)	β	SE(β)	β	SE(β)	β	SE(β)	β	SE(β)	β	SE(β)	β	SE(β)	β	SE(β)	β	SE(β)	β	SE(β)
**Internal/ external rotation in the GHJC**	**M_{max} [Nm/kg]**	0.069	0.003	0.019	0.003	-0.011	0.003	-0.009	0.003	0.006	0.003	-0.023	0.003	0.008	0.005	0.008	0.004	0.009	0.005	0.005	0.004
	**RoM [°]**	68.943	4.852	0.184	3.781	24.724	3.874	23.997	3.781	6.862	3.781	43.592	3.781	-4.033	5.413	-3.605	5.347	-0.843	5.413	0.827	5.347
	**$\alpha(M_{max})$ [°]**	-42.958	2.027	8.045	2.176	29.814	2.230	29.420	2.176	12.301	2.176	36.134	2.176	5.264	3.116	4.110	3.078	0.555	3.116	14.777	3.078
**Adduction/ abduction in the GHJC**	**M_{max} [Nm/kg]**	0.103	0.006	0.003	0.007	-0.020	0.007	-0.007	0.007	0.021	0.007	0.038	0.007	-0.009	0.010	-0.009	0.010	-0.004	0.010	-0.008	0.010
	**RoM [°]**	13.632	2.626	0.546	3.120	-3.307	3.196	-3.367	3.120	-0.483	3.120	76.749	3.120	-0.613	4.467	-2.239	4.412	-1.612	4.467	4.740	4.412
	**$\alpha(M_{max})$ [°]**	-53.492	2.646	-1.742	2.648	41.913	2.714	40.752	2.648	-0.145	2.648	#####	2.648	-0.403	3.792	0.482	3.745	-1.775	3.792	1.774	3.745

Table S4: Random effects coefficients and standard error (SE) for the linear mixed model of the external and internal rotation exercises

		external rotation exercise				internal rotation exercise			
		Residual		Intercept variance [subject = Subject]		Residual		Intercept variance [subject = Subject]	
		Estimate	SE	Estimate	SE	Estimate	SE	Estimate	SE
Internal/ external rotation in the GHJC	M_{max} [Nm/kg]	0.000	0.000	0.000	0.000	0.000	0.000	0.000	0.000
	RoM [°]	85.759	12.316	196.800	87.782	119.582	16.997	198.924	89.937
	$\alpha(M_{max})$ [°]	28.415	4.079	20.874	10.121	49.236	6.998	14.754	8.420
Adduction/ abduction in the GHJC	M_{max} [Nm/kg]	0.000	0.000	0.000	0.000	0.000	0.000	0.000	0.000
	RoM [°]	58.407	8.391	24.349	13.009	59.107	8.401	20.403	11.252
	$\alpha(M_{max})$ [°]	42.085	6.043	41.963	19.735	56.457	8.024	22.288	11.938
